# Examining racial and social vulnerability disparities in the outpatient treatment of uncomplicated cystitis at a Southern California Academic Hospital

**DOI:** 10.1017/ash.2023.469

**Published:** 2023-11-29

**Authors:** Caleb C. McLeod, Hadeel Al-Fayiz, Sasha Rodriguez, Karen K. Tan, Jacinda C. Abdul-Mutakabbir

**Affiliations:** 1 Department of Pharmacy Practice, Loma Linda University School of Pharmacy, Loma Linda, CA, USA; 2 Department of Pharmacy Services, Loma Linda University Medical Center, Loma Linda, CA, USA; 3 Division of Clinical Pharmacy, Skaggs School of Pharmacy and Pharmaceutical Sciences, University of California San Diego, San Diego, CA, USA; 4 Division of the Black Diaspora and African American Studies, University of California San Diego, San Diego, CA, USA

## Abstract

Racially and ethnically minoritized (REM) patients are disproportionately impacted by infectious diseases. In our study, REM patients were more likely to receive care for urinary tract infections in the emergency department or urgent care, were younger, and were more likely to have higher social vulnerability.

## Introduction

Infectious diseases are a major contributor to racial and ethnic mortality disparities in the United States, with inequities in social vulnerability (SV) factors (including poverty, minimal transportation access, and crowded housing) being considered key contributors.^1,2^ In areas populated by a majority of racially and ethnically minoritized (REM) individuals, these inequities may impact patients’ ability and perceived need to access health care. Differences in healthcare utilization inevitably translate to an increased reliance on higher acuity resources within REM communities, such as emergency departments (EDs) and urgent care (UC).^3^

Urinary tract infections (UTIs) are one of the leading outpatient indications for antimicrobial therapy, and while associated morbidity may be low, injudicious antimicrobial use represents a risk of inappropriate prescribing.^4,5^ There is a paucity of literature that explores racial, ethnic, and SV differences in the outpatient treatment of UTI.^4^ Here, we attempt to describe racial and SV differences in the utilization of ED and UC resources for the treatment of UTI, with a focus on uncomplicated cystitis, as a first step to improving prescribing and treatment practices in vulnerable communities.

## Methods

### Study design, patient population, and location

This retrospective, observational study evaluated adult patients with a diagnosis of cystitis treated in the ED and two UC centers associated with Loma Linda University Health from January 1, 2021 to April 30, 2021. Patients were included if they had a urine culture collected in the ED or UC and were diagnosed with acute cystitis by International Classificaiton of Disease (ICD-10) code N30. Patients were dichotomized to REM and non-racially and ethnically minoritized (n-REM) groups based on patient-reported race and ethnicity. REM groups include self-identified race as Black or African American, Asian or Pacific Islander, Middle Eastern, or Hispanic origin or Latin race as well as self-identified ethnicity of Hispanic or Latino, and the n-REM group only included self-identified race as White with an ethnicity not defined as Hispanic or Latino. This study was approved by the institutional review board of Loma Linda University.

### Data collection and study definitions

Patient demographic information and comorbid conditions were collected by chart review. Urine culture microbiologic results were recorded only for initial cultures collected in the ED or UC. Admission and discharge dates were collected, and a length of stay longer than 2 days was selected to identify patients likely requiring inpatient admission. Antibiotic susceptibility was defined by CLSI M100. Encounter antimicrobials were defined only as antimicrobials given during ED or UC encounter. Discharge antimicrobials were recorded.

### Social Vulnerability Index (SVI)

SV was estimated utilizing the CDC SVI tool, and it is calculated by assigning scores to 16 social factors and grouped into 4 categories: socioeconomic status, household composition, and disability, minority status and language, and housing type and transportation.^6^

### Statistical analysis

Data were collected and managed using REDCap version 13.1.29 software (Vanderbilt University, Nashville, TN) electronic data capture tools hosted by Loma Linda University. Data analysis was performed using SPSS software version 27 (IBM, Armonk, NY). Categorical variables were reported as numbers and percentages. Continuous data were assessed for normality using the Shapiro–Wilk test and reported as either means with standard deviation or medians with interquartile ranges. Data were compared between n-REM and REM groups using χ^2^ or Fisher’s exact tests for categorical variables and the Student *t* test or Mann–Whitney test for continuous variables. Statistical significance was defined as *P* ≤ 0.05.

## Results

### Study population

During the study period, 250 patients with either an ED or UC encounter with a diagnosis of acute cystitis were screened, and 114 REM patients and 73 n-REM patients were included; patients identifying as female were most represented (151/187; 87%), and most patients were overweight (BMI 25–29.9, *n* = 56/187, 33%) or obese (BMI 30–34.9, *n* = 32/187, 19%). In the REM group, 79 patients (69%) identified as Hispanic or Latino, 20 patients identified as Black non-Hispanic (18%), and 13 (11%) identified as Asian. REM patients were significantly younger than n-REM patients (median age 47 vs 67 years, *p* < 0.001). Despite differences in age, there was no statistically significant difference in comorbidities between REM and n-REM groups, with similar rates of diabetes and chronic kidney disease (CKD). REM patients were also significantly more likely to be at the highest level of SV group when compared to n-REM patients (72% vs 44%, *p* <0.001) (Table 1).

**Table 1.** Demographic, microbiological, and treatment data among REM and n-REM patients with uncomplicated cystitis

Characteristic	REM (*n* = 114)	n-REM (*n* = 73)	Total (*n* = 187)	*P*
Age, median, years (IQR)	47 (31–66)	67 (43–78)	59 (33–73)	<0.001
Sex, female, *n* (%)	90 (79%)	61 (84%)	151 (81%)	0.280
Race and ethnicity, *n* (%)				
White, non-Hispanic		73 (100%)		
Hispanic/Latino	79 (69%)			
Black/African American and non-Hispanic	20 (18%)			
Asian	13 (11%)			
Middle Eastern	2 (2%)			
Social vulnerability, *n* (%)				
Low	1 (1%)	2 (3%)	3 (2%)	0.561
Moderate	15 (13%)	20 (27%)	35 (19%)	0.021
Moderate-high	16 (14%)	18 (25%)	34 (18%)	0.081
High	81 (72%)	32 (44%)	113 (61%)	<0.001
BMI, *n* (%)*				
<18.5	5 (5)	1 (1)	6 (4)	0.407
18.5-24.9	24 (24)	22 (32)	46 (27)	0.168
25-29.9	33 (33)	23 (34)	56 (33)	0.745
30-34.9	19 (19)	13 (19)	32 (19)	0.845
35-39.9	9 (9)	4 (6)	13 (8)	0.769
≥40	10 (10)	5 (7)	15 (9)	0.785
Comorbidities, *n* (%)				
Diabetes	33 (29%)	19 (26%)	52 (28%)	0.739
Chronic Kidney Disease (CKD)	13 (11%)	12 (16%)	25 (13%)	0.380
Malignancy	12 (11%)	4 (5%)	16 (9%)	0.290
Chronic Foley catheter	9 (8%)	7 (10%)	16 (9%)	0.790
Presented to ED or UC, *n* (%)				0.874
ED	77 (68%)	48 (66%)	125 (67%)	
UC	37 (32%)	25 (34%)	62 (33%)	
Primary urinary pathogen, *n* (%)^†^	*n* = 97	*n* = 62	*n* = 159	0.305
*Escherichia coli*	41 (43%)	23 (37%)	64 (40%)	
*Klebsiella* species	5 (5%)	8 (23%)	13 (8%)	
*Proteus* species	1 (1%)	3 (5%)	6 (4%)	
Mixed flora	33 (34%)	19 (31%)	52 (33%)	
Initial encounter antimicrobial, *n* (%)				
Intravenous beta-lactam	42 (37%)	21 (29%)	63 (34%)	
Ceftriaxone	37 (32%)	14 (19%)	51 (27%)	
Oral beta-lactam	8 (6%)	4 (5%)	12 (6%)	
Nitrofurantoin	1 (1%)	3 (4%)	4 (2%)	
Other	8 (7%)	11 (15%)	19 (10%)	
No antimicrobial	55 (48%)	34 (47%)	89 (48%)	
Discharge antimicrobial, *n* (%)				
Oral beta-lactam	62 (54%)	40 (55%)	102 (55%)	
Cephalexin	59 (52%)	39 (53%)	98 (52%)	
Nitrofurantoin	23 (20%)	13 (18%)	36 (19%)	
Fluoroquinolone	7 (6%)	3 (4%)	10 (5%)	
Trimethoprim/sulfamethoxazole	6 (5%)	3 (4%)	9 (5%)	
Other	8 (7%)	3 (4%)	11 (6%)	
No discharge antimicrobial	8 (7%)	11 (15%)	19 (10%)	
Discharge antimicrobial activity, *n* (%)				0.689
Yes	38 (48%)	31 (55%)	69 (51%)	
No	29 (37%)	17 (30%)	46 (34%)	
Not reported	12 (15%)	8 (14%)	20 (15%)	
Length of stay >2 days, *n* (%)	18 (16%)	18 (25%)	36 (19%)	0.183
1-year all-cause readmission, *n* (%)	61 (54%)	34 (47%)	95 (51%)	0.372
Readmission due to UTI, *n* (%)	16 (14%)	12 (16%)	28 (15%)	0.678

* BMI was unable to be calculated for 14 REM patients and 5 n-REM patients who did not have documented height and/or weight.

† Primary urinary pathogen reported based on 159 patients (97 REM and 62 n-REM) with growth on urine cultures.

### Microbiology and treatment

The most commonly isolated primary organism was *Escherichia coli* (*n* = 80, 43%) followed by *Klebsiella spp.* (*n* = 15, 8%), and 55 (29%) patients had only mixed flora isolated on urinary cultures. Ceftriaxone was the most common antimicrobial administered during EC or UD encounters, while 89 patients (48%) received no antimicrobials during their encounters. On discharge, 168 patients (89%) were prescribed oral antibiotics, most commonly cephalexin (*n* = 98, 52%) or nitrofurantoin (*n* = 35, 19%), consistent with local antimicrobial susceptibility. REM patients were less likely to have documented susceptibility (48% vs 55%) and more likely to have documented resistance (37% vs 30%) to the agent prescribed on discharge when compared to n-REM patients, although these differences were not statistically significant (*p* = 0.689). Only eight patients across both groups had antimicrobials adjusted after discharge when culture susceptibilities resulted. REM patients were also less likely to have a length of stay longer than 2 days when compared to n-REM patients (16% vs 25%, *p* = 0.183), although this difference was also not statistically significant.

## Discussion

In this study, our findings identified no significant differences in the treatment approaches or outcomes between these REM and n-REM groups. However, notable demographic differences were appreciated, including age differences in comorbidities which may contribute to the increased likelihood of required readmission and significant SVI disparities in the REM cohort. These results denote a potential area for the development of ED- or UC-specific clinical pathways for REM patients who present with suspected UTIs to hospital centers that primarily serve vulnerable communities. Recognizing the significance of age as a potential factor in healthcare disparities, efforts can be directed toward tailoring educational materials and interventions specifically for this demographic. By considering the unique needs and health literacy of this population, healthcare providers and organizations can potentially engage patients in lifestyle changes to prevent uncomplicated cystitis.

Microbiological distribution of uropathogens was similar between REM and n-REM patients; however, REM patients were more likely to have been prescribed antimicrobials on discharge with documented inactivity against their uropathogen, which may have collateral effects and result in a need for continued ED and UC utilization and incurred costs.^7,8^ These study results highlight opportunities to improve both institutional empiric and culture-directed antimicrobial use.

This study has several limitations to consider. The retrospective nature of this study and reliance on ICD-10 diagnosis, rather than clinical presentation, likely limits the accuracy of UTI diagnosis, although these diagnosis codes likely indicate provider perception. It is important to note that the study aimed to describe differences between REM and n-REM patients with UTI, and outcomes were not available for comparison. Assessments of health literacy were also missing, which is a recognized independent predictor of health behaviors and could further impact outcomes.^9^ Finally, our institution serves a predominantly Hispanic population within an area of high SV. To gain a more comprehensive understanding of health disparities in uncomplicated cystitis, future studies should include patients from low to moderate SVI and evaluate the impact of both deprivation and health literacy on clinical outcomes.
